# Arctigenin as a promising anticancer agent: a comprehensive review of molecular mechanisms across major carcinomas

**DOI:** 10.3389/fphar.2026.1794520

**Published:** 2026-06-24

**Authors:** Deena Elsori, Pratibha Pandey, Mohammad Abohassan, Mohd Saeed, Nida Khan, Sorabh Lakhanpal, Meenakshi Verma, Anuj Kumar Rana, Fahad Khan

**Affiliations:** 1 Faculty of Resilience, Rabdan Academy, Abu Dhabi, United Arab Emirates; 2 University Centre for Research and Development, Chandigarh University, Mohali, Punjab, India; 3 Department of Clinical Laboratory Sciences, College of Applied Medical Sciences, King Khalid University, Abha, Saudi Arabia; 4 Health and Medical Researches Centre, King Khalid University, Abha, Saudi Arabia; 5 Department of Biology, College of Science, University of Hail, Hail, Saudi Arabia; 6 Department of General Medicine, Faculty of Medicine and Health Sciences, Integral Institute of Medical Sciences & Research, Integral University, Lucknow, India; 7 School of Pharmaceutical Sciences, Lovely Professional University, Phagwara, Punjab, India; 8 School of Applied and Life Sciences, Uttaranchal University, Dehradun, India; 9 Department of Community Medicine, Saveetha Medical College and Hospitals, Saveetha Institute of Medical and Technical Sciences, Chennai, Tamil Nadu, India

**Keywords:** anticancer, arctigenin, bioavailability, lignan, phytochemical

## Abstract

Despite the presence of numerous efficacious bioactive compounds, the development of effective cancer therapies remains necessary. Thus, the exploitation of the medicinal potential of bioactive compounds in cancer management has become an urgent demand. Arctigenin (AG), a natural lignin derived from *Arctium lappa*, not only suppresses the proliferation of diverse cancerous cells, including those in the liver, stomach, lungs, and colon, but also modulates various antitumor, intracellular, antioxidative, and anti-inflammatory activities. Arctigenin has also been shown to overcome tumor resistance via cell-cycle arrest, activation of apoptotic pathways, caspase activation, and proteasome regulation. It also downregulates numerous factors implicated in tumor migration and angiogenesis, including VEGF, matrix metalloproteinases, TGF-β, and N-cadherin. The restricted solubility of arctigenin in water indicates that modifying this compound through amino acid esterification may enhance its pharmacogenetic properties. The use of arctigenin or its derivatives may facilitate the development of novel chemotherapeutic strategies for future therapies. This review identifies common therapeutic targets and cancer-specific responses by comparing mechanistic insights across prostate, cervical, pharyngeal, and other carcinomas. Arctigenin is positioned as a viable option in anticancer research through a critical assessment of current limitations in preclinical and clinical research and by providing future perspectives on translational potential, drug development techniques, and combination therapies. Although there are publications that summarize the mechanism of action of arctigenin, none have explicitly elaborated on its anticancer mechanism across various carcinomas.

## 1 Introduction

Nature contains a plentiful array of physiologically bioactive compounds. A large percentage of the chemotherapeutic medications currently in use are derived from natural sources. Thus, in recent decades, bioactive compounds such as polyphenols have been used to treat numerous human malignancies. Several studies have confirmed the medicinal potential of phytochemicals for cancer therapy (Rudzińska et al., 2023). The anticancer potential of phytochemicals lies in their ability to modulate apoptosis and autophagy as therapeutic mechanisms (Ahmed et al., 2024). Previous reports have focused on bioactive dietary flavonoids found in foods as promising novel drug discovery (Li et al., 2024; Liu J. et al., 2024). However, detailed reviews on the effects of polyphenols against numerous carcinomas have not yet been updated in the literature. Polyphenols are a class of phytochemicals predominantly present in plant-based foods (vegetables, whole grains, and seeds) (Omoruyi et al., 2024). Lignans (phenolic compounds) are commonly found in plants such as Linum, Sesamum, and Forsythia. The precursors of lignans are metabolized into enterodiol, enterolactone, and other lignan metabolites. Arctigenin (AG), a bioactive dibenzylbutyrolactone lignan reported in Chinese herbal medicine, has gained considerable attention over the past decade (Balkrishna et al., 2024). Burdock, which contains AG, is a member of the Asteraceae family and is frequently used to treat viral illnesses, such as a sore throat. Lignans display considerable solubility in organic solvents but little to negligible solubility in water. According to Umezawa (2003), these compounds can be classified into eight categories: arylnaphthalene, dibenzylbutane, furan, aryltetralin, furofuran, dibenzylbutyrolactol, dibenzylbutyrolactone, and dibenzocyclooctadiene. AG may target multiple intracellular pathways implicated in tumor therapy and modulate both forms of apoptotic cell death in tumor cells.

AG has also been reported to modulate the cell cycle by regulating the production of cyclins and CDKs. Moreover, AG expedites cellular senescence in gallbladder cancer by blocking the RAF–MEK–ERK signaling pathway (Zhang et al., 2017). Furthermore, modulation of metastatic protein expression (including MMP and EMT-related factors) augments the anticancer efficacy of AG against tumor invasion and migration. AG regulates several biological responses, including signal transduction pathways. Examining the mechanisms of action of bioactive metabolites would enhance our understanding of tumor biology and expedite advancements in the field of innovative anticancer therapies. A diverse array of molecular mechanisms has been shown to elucidate the pharmacological efficacy of AG, prompting us to summarize its anticancer potential in two of the most lethal carcinoma groups: breast and gastrointestinal malignancies. Furthermore, synergistic effects and toxicological findings were examined. Numerous signaling pathways, such as cell-growth arrest, ferroptosis, and autophagy, are discussed and detailed in relevant sections describing its role in several carcinomas. We also clarify whether arctigenin acts through these pathways or cancer-specific mechanisms.

## 2 Search strategy

Literature regarding the anticancer mechanism of AG was obtained from online databases, such as SciFinder, Scopus, PubMed, and Web of Science, up to August 2025. Clinicaltrials.gov was used to identify registered clinical trials involving AG. The search terms employed were “arctigenin,” “cancer,” “anticancer,” and “pharmacokinetics”.

## 3 Bioavailability and pharmacokinetics of arctigenin

The intestinal and circulatory metabolism of AG may affect its biological action. In response, further research was conducted on AG metabolites from the intestinal microbiota and hepatic metabolism. The revised text includes a brief review of plasma and gastrointestinal metabolites and their potential pharmacological effects. However, the biological activity of these metabolites is limited; mechanistic insights are still needed. LC–MS/MS studies have monitored arctigenin and its major metabolites (such as arctigenic acid and arctigenin-4′-O-glucuronide) in plasma, demonstrating that these metabolites circulate following intestinal absorption. Evidence has shown extensive first-pass metabolism in the intestine. Pharmacokinetic modeling studies have also shown that only metabolites such as arctigenic acid and glucuronide conjugates are detected in the blood following oral administration, indicating near-complete presystemic metabolism. The main metabolic processes are glucuronidation and hydrolysis in the gut and liver (Gao et al., 2014a).

The pharmacokinetic properties of AG have been widely investigated in various animal models. Despite its remarkable intestinal absorption, the plasma concentration of AG remains remarkably low after oral administration (Gao et al., 2018). The mechanism behind these low concentrations is the significant first-pass metabolism of AG in the liver and colon, where it is conjugated with glucuronic acid (Gao et al., 2014b; Ikeda et al., 2016). Furthermore, AG was rapidly distributed throughout the tissues following intragastric administration. In addition to the liver and colon, AG was detectable in the brain and bone marrow after subcutaneous injection (He et al., 2013; Li et al., 2017). The widespread distribution of AG across various organs underpins its therapeutic potential in multiple human tissues and organ systems.

Prolonged excretion resulting from enterohepatic and intrahepatic circulation leads to enhanced tissue retention (He et al., 2019). Consequently, oral administration may represent a more effective method for AG delivery, providing enhanced bioavailability. Furthermore, molecular modifications may improve its low aqueous solubility and thereby overcome the restricted bioavailability of AG. The valine ester derivatives of AG exhibit 6–8 times higher bioavailability than pure AG, leading to significantly enhanced antitumor efficacy. The solubility of AG can be further augmented by incorporating hydroxyl groups, thereby enhancing its anti-T. gondii potency (Cai et al., 2018; Zhang et al., 2018).

AG toxicity has been evaluated in both humans and animals. Subcutaneous treatment of beagle dogs with AG (60 mg/kg daily) for 28 days led to systemic toxic effects, such as hepatic damage, leukocytosis, and renal failure. A minimal dose of AG (6 mg/kg) does not induce significant deleterious effects (Li et al., 2019a). Four-week oral treatment with AG (3–12 g, once daily) resulted in no dose-limiting toxicities in humans; however, moderate side effects, including elevated bilirubin, transaminase, hyperglycemia, and plasma lactate levels, were noted (Fujioka et al., 2018; Ikeda et al., 2016). Specifically, our manuscript now provides a more cohesive overview of key signaling pathways, highlighting the interactions between cell signaling processes such as and cell-cycle regulation, apoptosis, and autophagy. We have also included additional details to better distinguish common mechanisms from cancer-type-specific mechanisms of arctigenin action. However, AG remains in the research stage and has, therefore, not been established as a standard therapy.

## 4 Breast cancer and arctigenin

Despite the medicinal potential of phytoestrogens in mitigating the risks of menopause, osteoporosis, and cardiovascular disease, their protective effects against breast cancer remain unclear. Arctigenin inhibits the growth of breast cancer cells, including both hormone-positive and triple-negative types, through multiple mechanisms, including anti-proliferative effects, ferroptosis induction, suppression of metastasis and apoptosis, and modulation of cell signaling pathways. A previous study examined the anticancer effects of arctigenin on the metastasis of human MCF-7 and MDA-MB-231 breast cancer cells. In ER-positive MCF-7 cells, arctigenin effectively suppressed tumor promoter TPA-induced cell migration and invasion. Arctigenin demonstrated comparable anti-metastatic effects in ER-negative MDA-MB-231 cells, indicating that its anti-metastatic properties operate through the inhibition of MMP-9 and uPA (human urokinase-type plasminogen activator) via the AKT, NF-κB, and MAPK signaling pathways in breast cancer. Consequently, arctigenin supplementation may be beneficial for patients with breast cancer (Maxwell et al., 2017).

A separate study showed that AG markedly suppresses the invasion of MDA-MB-231 cells by downregulating MMP-2, MMP-9, and heparanase (Lou et al., 2017). Obesity is the primary contributor to several types of breast cancer. Arctigenin inhibits adipogenesis in pre-adipocytes and induces apoptosis in MCF-7 breast cancer cells by regulating β-catenin expression (Lee et al., 2017). AG impedes STAT3 binding to genomic DNA by disrupting the hydrogen bonds between DNA and STAT3. Arctigenin has been identified as a new STAT3 inhibitor and demonstrated considerable cytotoxic effects on triple-negative breast cancer (TNBC) cells. CIP2A is an intrinsic inhibitor of protein phosphatase 2A (PP2A) that enhances the migration and invasion of diverse cancer cells. Arctigenin suppresses triple-negative breast tumors by targeting CIP2A to reactivate protein phosphatase 2A. AG-induced prevention of metastasis was linked to the reactivation of PP2A, downregulation of CIP2A, and phosphorylation of AKT. CIP2A inhibition augments AG-induced suppression of metastasis and apoptosis in triple-negative breast cancers (Huang et al., 2017).

Multiple studies have indicated that AG is a phytoestrogen that induces proliferation by binding to estrogen receptors. A study investigated the effect of AG on MCF-7 (ERα-positive) human breast cancer cells to ascertain the safety of AG ingestion in patients with breast cancer. This study demonstrated that phytoestrogen AG primarily affects the mTOR pathway in ERα-positive MCF-7 human breast cancer cells, resulting in autophagy-mediated cell death and decreased ERα expression. The synergistic efficacy of AG and tamoxifen indicated that AG consumption is not only safe for patients with hormone-sensitive malignancies but may also serve as an effective adjunctive treatment (Maxwell et al., 2018).

A recent study demonstrated that AG significantly inhibits cell migration and invasion in 4T-1 mouse TNBC cells by suppressing MMP-9 activity and metastasis-promoting factors (MMP-9, MMP-3, and COX-2) through a downregulated MAPK/AP-1 signaling pathway (Lee M. G. et al., 2020). AG also diminished the promoter activities of TSLP and GM-CSF by inhibiting NF-κB p65 nuclear translocation. AG-induced reduction of TSLP and GM-CSF led to the suppression of STAT3 phosphorylation and β-catenin signaling, which, in turn, diminished the invasion and stemness of breast cancer cells. These findings indicated that AG is a viable candidate for cytokine-targeted breast cancer therapy (Shi et al., 2020). Another study provided a novel approach for the development of targeted therapies for breast cancer. A separate study reported the effects of AG on 4EBP1 (eukaryotic translation initiation factor 4E binding protein 1) in breast cancer cells. AG impedes the migration and invasion of human breast cancer cells by targeting 4EBP1 (Luo et al., 2021).

Human epidermal growth factor receptor 2 (HER2) is a key therapeutic biomarker of breast carcinoma (Jang et al., 2025). AG administered to HER2-overexpressing SK-BR-3 cells diminished cell viability and inhibited the HER2/EGFR1 signaling pathway. Moreover, AG-enhanced H_2_AX phosphorylation was observed, whereas RAD51 and survivin were downregulated, indicating that AG impaired DNA repair mechanisms and promoted DNA damage. AG treatment induced caspase-7 and PARP cleavage via the accumulation of mitochondrial cytochrome c in the cytoplasm. In AG-treated cells, LC-3 and SQSTM1/P62 levels were elevated through the AKT/mTOR and AMPK signaling pathways (Lee et al., 2021).

**FIGURE 1:**
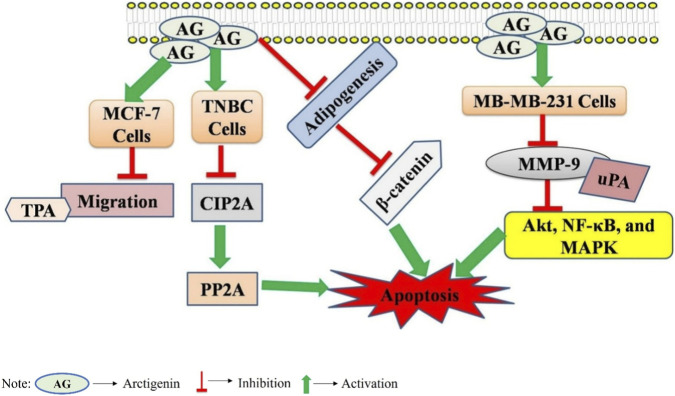
Diagrammatic illustration of the efficacy of arctigenin in breast carcinoma by targeting migration and invasion, angiogenesis, and altered signaling pathways, ultimately leading to apoptotic cell death. Abbreviations: CIP2A, cancerous inhibitor of protein phosphatase 2A; TPA, tissue plasminogen activator; CIP2, Csx1-interacting protein 2; MMP, matrix metalloproteinase; AKT, AK strain transforming; MAPK, mitogen-activated protein kinase; TNBC, triple-negative breast cancer; uPA, urokinase-type plasminogen activator.

The increase in drug resistance necessitates the prompt identification of novel therapeutic agents, notwithstanding the availability of various targeted treatments. Experimental findings indicated that AG did not trigger apoptosis in ER^+^ cells; instead, it caused G1 phase arrest by reducing cyclin D1 levels without affecting CDK4/6 levels. Furthermore, AG treatment reduced cyclin D1 levels by inducing AKT/GSK3β-mediated degradation (Zhu et al., 2020; Thilagavathi et al., 2023). Three primary markers were used to categorize breast cancer: ER, HER2, and PR. AG induces cell death by initiating apoptosis in both ER-positive and ER-negative breast cancer cells (Luo et al., 2021). In triple-negative breast cancer (TNBC), AG has shown potential by inhibiting STAT3 signaling, reducing tumor growth, and suppressing survival pathways. These findings support the potential application of arctigenin as a therapeutic agent for the treatment of breast cancer, including ER-positive and triple-negative subtypes (Figure 1) (Yoo et al., 2024).

## 5 Gastrointestinal cancer

### 5.1 Liver cancer and arctigenin

Liver carcinoma has long been associated with high mortality in both men and women. Its progression is linked to chronic liver disorders, while late detection and therapy resistance limit outcomes. AG has been reported to inhibit cancer cell proliferation, induce apoptosis, and modulate signaling pathways (such as NF-κB and PI3K/AKT). However, clinical validation of its therapeutic efficacy is lacking (Jiang et al., 2015). AG has been widely documented to exhibit significant biological activities, including anticancer, anti-inflammatory, and antiviral effects. AG induced apoptosis in SMMC7721 and HepG2 cancer cells, with a more pronounced effect in HepG2 cells than in SMMC7721 cells. Moreover, AG treatment resulted in the loss of MMP, Bax upregulation, Bcl-2 downregulation, cytochrome c release, caspase-9/3 activation, and poly (ADP-ribose) polymerase cleavage in both SMMC7721 and HepG2 cells, indicating that AG-induced apoptosis is linked to the mitochondrial-mediated pathway. Furthermore, activated caspase-8 and elevated Fas/FasL and TNF-α expression levels indicated significant involvement of the Fas/FasL-related pathway in this mechanism. In addition, AG-induced apoptosis was associated with inactivation of the PI3K/p-AKT pathway, enhanced p53 protein accumulation, and suppressed NF-κB nuclear translocation, leading to increased sensitivity of HepG2 cells compared with SMMC7721 cells (Lu et al., 2015).

Another study reported increased apoptosis and activation of caspase 3/9 in AG-treated HCC cells. Combination therapy with AG and LY294002 (a PI3K inhibitor) markedly enhanced apoptosis. AG treatment counteracted several processes associated with cancer progression, such as reduced Mcl-1, survivin, and Bcl-xL levels, through inhibition of the AKT pathway. This study demonstrated a significant anticancer effect of AG in HCC cells via the inactivation of the PI3K/AKT signaling pathway (Jiang et al., 2015). Another study examined the antimetastatic efficacy of AG in a lung cancer mouse model. AG treatment inhibited TGF-β-induced alterations in metastatic morphology and cellular migration. AG reduced the phosphorylation and transcriptional activation of TGF-β-induced Snail and Smad2. AG decreased the expression of N-cadherin and increased that of E-cadherin by reducing the nuclear translocation of phospho-Smad2/3. Furthermore, AG inhibited TGF-β-induced β-catenin transcription and ERK phosphorylation (Xu et al., 2017).

AG treatment further suppressed proliferation of both HepG2 and Hep3B cell lines by directly binding C/EBPα to the gankyrin promoter. PPARα interacts with C/EBPα, resulting in a negative regulatory effect on gankyrin expression. This study identified a novel mechanism of action for arctigenin in inhibiting liver cancer cell proliferation (Sun et al., 2018). Subsequent research examined the anti-metastatic properties and mechanisms of AG in hepatocellular carcinoma, revealing that AG demonstrated considerable cytotoxicity toward HepG2 and SMMC 7721 cells. Furthermore, AG reduced the GSK3β-dependent WNT/β-catenin signaling pathway. Moreover, AG may impede the epithelial–mesenchymal transition by enhancing the expression of epithelial markers and reducing the expression of mesenchymal markers. Intraperitoneal administration of AG significantly suppresses the growth of subcutaneously implanted tumors and markedly reduces liver metastasis (Lu et al., 2019).

**FIGURE 2:**
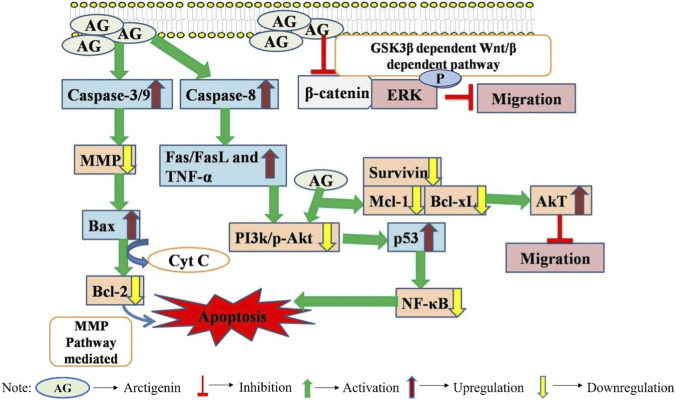
Possible mechanisms of arctigenin against liver carcinoma through the modulation of numerous cellular and molecular components of signaling pathways. Abbreviations: CytC, Cytochrome C; MMP, matrix metalloproteinase; ERK, extracellular signal-regulated kinase; Bax, Bcl-2-associated X protein; AKT, AK strain transforming; TNF, tumor necrosis factor.

In HUH-6 cells, AG demonstrated its antitumor effect by triggering apoptosis through TNFR1, which recruits Complex IIa to activate caspases 8 and 3/7. These findings may aid the development of therapeutic drugs for hepatoblastoma (HUH-6 cells) (Naoe et al., 2019). *Arctium lappa* and *Forsythia suspensa* fruit extracts significantly inhibited HepG2 cell proliferation and autophagy. AG (a bioactive lignin) decreased HepG2 cell growth and the expression levels of autophagy-related proteins, indicating that AG might inhibit the autophagy process, resulting in the accumulation of sequestosome 1/p62 (p62). AG did not influence caspase-3 activation or PARP cleavage, indicating that the antiproliferative effect of AG may occur independently of apoptosis. Consequently, the findings of this study substantiate the potential of AG in the formulation of drugs for autophagy research and cancer chemoprevention (Okubo et al., 2020) (Figure 2).

### 5.2 Colorectal carcinoma and arctigenin

**FIGURE 3:**
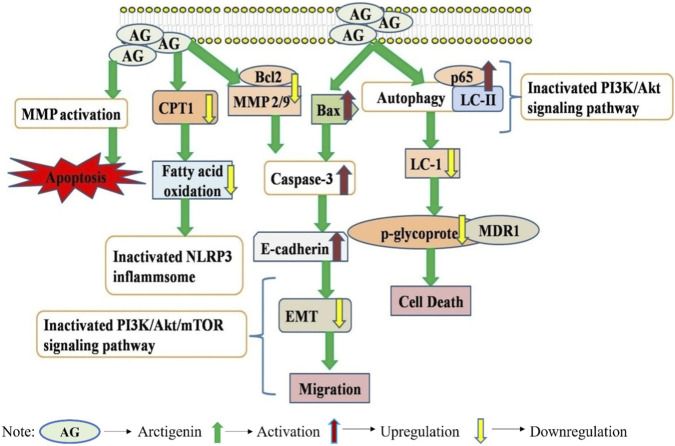
Possible mechanisms of arctigenin against colorectal carcinoma through the modulation of numerous cellular and molecular components of signaling pathways. Abbreviations: CytC, Cytochrome C; MMP, matrix metalloproteinase; NLRP, nucleotide-binding oligomerization domain; ERK, extracellular signal-regulated kinases; Bax, Bcl-2-associated X protein; AKT, AK strain transforming; TNF, tumor necrosis factor; CPT1, carnitine palmitoyltransferase I; Lic1, leucine-rich repeat protein; EMT, epithelial–mesenchymal transition; MDR1, multidrug-resistance (MDR1) P-glycoprotein.

The antimetastatic effects of AG on colorectal metastasis indicate its potential as a prospective therapeutic agent. AG triggered cell-cycle arrest and apoptosis in CT26 cells through the intrinsic apoptotic pathway via MAPK activation. In many metastatic phenotypes, AG regulates EMT by enhancing the expression of epithelial marker E-cadherin and reducing the expression of mesenchymal markers. Furthermore, AG impedes invasion and migration by reducing the expression of MMP-2/9 in colorectal cancer (Han et al., 2016). Both colorectal cancer cell lines (SW480 and SW620) were used to examine the effects of AG on R-SW480 and R-SW620 (cisplatin-resistant colorectal cancer cell lines). AG markedly suppressed cell growth in both R-SW480 and R-SW620 cells compared with that in cisplatin-treated colorectal cells. AG enhanced cell death and expression of the pro-apoptotic protein cleaved-caspase-3/9 in both R-SW480 and R-SW620 cells. Furthermore, AG enhanced autophagy and the expression levels of p65 and LC3-II and, while suppressed LC3-I levels. AG reduced the inhibitory efficacy of cisplatin, oxaliplatin, doxorubicin, and paclitaxel in both colorectal cell lines. Furthermore, AG treatment dramatically decreased MDR1 mRNA and P-gp protein expression. AG sensitizes colorectal cancer cells via autophagy activation, inducing cell death, which may be particularly beneficial for adjuvant therapy (Wang et al., 2019) (Figure 3).

Additionally, AG-mediated suppression of colitis progression and colon carcinogenesis was observed in mice subjected to oral administration of azoxymethane (AOM) and dextran sulfate sodium (DSS)-induced colitis-associated cancer (CAC). AG suppresses the production of carnitine palmitoyltransferase 1 (CPT1), decreases α-tubulin acetylation, and interferes with NLRP3 complex assembly, thereby inactivating NLRP3 inflammasomes. Downregulation of fatty acid oxidation (FAO) inhibits NLRP3 inflammasome assembly in macrophages, thereby enhancing the protective effect of arctigenin against CAC. These findings underscore the potential efficacy of arctigenin in mitigating CAC risk in patients with colitis (Qiao et al., 2020). The expression of N-cadherin, a cell adhesion molecule, is typically increased in malignancies and is postulated to be a significant mediator of EMT, a process involved in metastasis. AG impeded cholangiocarcinoma advancement by modulating cell migration and survival through the N-cadherin and apoptotic pathways in cholangiocarcinoma (KKU-213A and KKU-100) cell lines (Janthamala et al., 2021).

A separate study demonstrated the inhibitory effects of AG on CRC cell growth and invasion, resulting in the activation of apoptosis. AG markedly reduced the expression levels of PCNA, Bcl-2, and MMP-2/9 and the increased expression levels of Bax and cleaved caspase-3 in CRC cells. Furthermore, AG suppressed EMT by enhancing E-cadherin expression and decreasing mesenchymal marker expression. Moreover, AG suppressed activation of the PI3K–AKT–mTOR signaling pathway, which is associated with cancer growth. *In vivo* studies have shown that AG markedly reduces tumor volume and size relative to the control group without causing notable deleterious effects on the liver (Chen et al., 2024). AG-treated gallbladder cancer cells exhibited reduced expression levels of the epidermal growth factor receptor (EGFR). Examination of the downstream kinase activity of EGFR indicated substantial inhibition of the RAF–MEK–ERK signaling cascade. AG significantly suppressed the proliferation of tumor xenografts, which is associated with EGFR downregulation and cellular senescence. Consequently, AG may cause senescence of GBC cells by altering the EGFR pathway. These observations indicate that EGFR is a crucial regulator of AG-induced senescence of gallbladder cancer (Zhang et al., 2017). AG inhibits cell proliferation, induces apoptosis, and suppresses invasion and metastasis. This is achieved by modulating signaling pathways, such NF-κB, PI3K/AKT, and STAT3, and regulating oxidative stress and cell-cycle arrest. AG has promising multi-target potential against colorectal cancer, but more clinical trials are needed to confirm its therapeutic use.

## 6 Lung cancer and arctigenin

TMEM16A is integral to physiological processes and may function as a therapeutic target for several illnesses. TMEM16A has recently emerged as a potential primary target in lung cancer. AG has been reported to prevent the growth of lung adenocarcinoma (LA) by suppressing TMEM16A, both *in vitro* and *in vivo*. AG inhibits the proliferation and migration of LA795 cells. Moreover, AG administered in a xenograft mouse model demonstrated substantial antitumor efficacy without any side effects. Consequently, the inhibitory potential of AG on LA cells results from the suppression of the MAPK pathway, thereby positioning AG as a promising lead candidate for the development of therapeutic agents for lung cancer through the targeting of TMEM16A (Guo et al., 2020).

AG treatment substantially inhibited the growth of A549/DDP cells in a separate experiment. The combination therapy was more efficacious in reversing the resistance of A549/DDP cells than monotherapy. The expression level of PTEN increased with increasing AG concentration, but STAT3 protein expression decreased with increasing AG concentration. The ADP group showed elevated PTEN and reduced STAT3 expression levels. AG modulates drug resistance in A549/DDP cells, potentially by a mechanism that reduces A549/DDP cell sensitivity to DDP, thereby influencing the stress pathways related to PTEN and STAT3. The amalgamation of AG and DDP significantly reduces the resistance of A549/DDP cells (Li et al., 2019b). AG inhibited lung cancer cell growth, induced apoptosis, and suppressed tumor invasion and metastasis. This is achieved by modulating signaling pathways, regulating oxidative stress, and controlling cell-cycle progression. AG has strong multi-target potential, but further clinical validation is needed to demonstrate its therapeutic value.

## 7 Pharyngeal carcinoma and arctigenin

The anticancer potential of AG was investigated using FaDu human pharyngeal carcinoma cells. AG promotes nuclear condensation, growth suppression, and fragmentation. While there is currently limited research on pharyngeal carcinoma specifically, studies on comparable head and neck malignancies indicate that arctigenin may prevent metastasis, induce apoptosis, and reduce tumor cell proliferation. AG-treated cells exhibited activation of caspase-3/7 expression and increased apoptosis. AG treatment resulted in elevated FasL (a death ligand associated with extrinsic apoptotic signaling pathways) expression, activation of caspase-8/9, and increased expression levels of p53 and BAX in FaDu cells. AG therapy led to reduced expression of Bcl-2 and Bcl-xL, which are integral components of the mitochondria-dependent intrinsic apoptotic pathway. Moreover, AG stimulates PARP and caspase-3 mediated cell death. AG also impeded the growth of FaDu cells via inhibition of the AKT, p38, and NF-κB signaling pathways. These results indicate that AG suppresses cell proliferation and triggers apoptosis (cell death) in FaDu (human pharyngeal cancer) cells via both intrinsic (mitochondria-mediated) and extrinsic (death receptor-mediated) pathways (Kang et al., 2022).

## 8 Cervical cancer and arctigenin

AG treatment decreased the viability of SiHa and HeLa cervical cancer cells. AG elevated cleaved-caspase 3 and E-cadherin expression and reduced the number of invading cells and vimentin and N-cadherin protein levels, thereby increasing apoptotic cell death. In SiHa cells, AG suppressed the FAK/paxillin pathway by upregulating FAK expression. The upregulation of FAK, which inhibits proliferation and invasion while promoting apoptosis, was counteracted by AG therapy. AG also inhibits growth and metastasis and promotes apoptosis *in vivo*. AG treatment consistently reduced p-FAK/FAK and p-paxillin/paxillin protein levels in the tumor tissues of xenografted mice (Liao et al., 2023). AG displayed growth inhibition of cervical cancer cells and tumors by modulating cell signaling pathways associated with inflammation and cell survival (Lee et al., 2022). AG has also demonstrated anti-metastatic potential. However, most evidence comes from *in vitro* and animal studies, and further clinical research is required to validate its safety in treating patients with cervical cancer.

## 9 Prostate cancer and arctigenin

Arctigenin played a vital role in preventing the growth and proliferation of prostate carcinoma cells. It has also disrupted androgen receptor signaling, which is required for metastasis of prostate carcinoma. Thus, further research should be conducted to elucidate its efficacy for use in the treatment of prostate cancer. Both Ag-treated LAPC-4 and LNCaP (androgen-dependent) prostate cancer cells displayed 10–20 times greater antiproliferative efficacy than quercetin. Furthermore, their combinatorial treatment synergistically enhanced the antiproliferative effect, exhibiting a more pronounced effect in wild-type LAPC-4 cells than in androgen receptor (AR) mutant LNCaP cells. AG exhibited significant potential to suppress AR protein expression in LAPC-4 cells. The combinatorial therapy markedly suppressed both the PI3K/AKT and AR pathways compared to the control group. The protein array also indicated that the combined treatment targeted several pathways, including the STAT3 pathway, in LAPC-4 cells. This combinatorial therapy further enhanced the suppression of cell migration in both cell lines relative to the assessment of individual drugs (Wang et al., 2015).

AG exhibits selective cytotoxicity toward acidity-resistant prostate cancer PC-3 cells via ROS-induced mitochondrial impairment and suppression of the PI3K/AKT/mTOR pathway (Lee et al., 2018). A separate study examined the mechanism of action of AG on acid-tolerant prostate cancer PC-3AcT cells in a lactic acid-enriched medium. AG, either independently or in combination with docetaxel, elicited substantial cytotoxicity in PC-3AcT cells compared to parental PC-3 cells. AG treatment resulted in a sub-G0/G1 peak in cell cycle, cell communication network factor 1 (CCN1) expression, elevated ROS levels, MMP loss, Annexin V-PE-positive fractions, and mitochondrial membrane depolarization, along with reduced phospho (p)-AKT levels and cellular ATP levels. These findings suggest a non-Warburg phenotype characterized by necroptosis through ROS-mediated CCN1 upregulation and mitochondrial damage (Lee Y. J. et al., 2020).

## 10 Other carcinomas

AG markedly inhibited proliferation and colony formation and induced apoptosis and S-phase cycle arrest in MG63 and U-2 OS cells. Additional research has indicated that AG impedes metastasis and facilitates cell cycle arrest and apoptosis in osteosarcoma cells by downregulating heme oxygenase-1 (HMOX1) (Xu et al., 2023). AG reduced invasiveness and motility in nasopharyngeal carcinoma (5-8F) cells. Consequently, treatment with AG decreased the expression of p-STAT3, EGFR, p-JAK2, and p-EGFR. These findings indicate that AG suppresses EGFR phosphorylation and decreases the levels of phospho-JAK2 and phospho-STAT3 (Huang et al., 2023). Arctigenin inhibits proliferation and induces death of B16-F10 melanoma cells, potentially by reducing the expression of BCL-2 and VEGF in these cells (Gao et al., 2024). Furthermore, AG induced notable growth inhibition, cell-cycle arrest (G0/G1), and activation of apoptosis in both U87MG and T98G human glioma cell lines. These findings necessitate further exploration of the anticancer properties of arctigenin in animal models of gliomas (Maimaitili et al., 2017) [Table 1].

**TABLE 1:** Summary of anticancer potential of AG against other carcinomas that are less explored.

Carcinoma	Target/Cell line/Animal model	Mode of action	Reference
Lung cancer	TMEM16A	Growth inhibition Suppression of the MAPK signaling pathway	Guo et al. (2020)
	A549/DDP cells	Increased PTEN expression Reduced STAT3 expression Modulated drug resistance	Li et al. (2019b)
Pharyngeal carcinoma	FaDu cells	Prompted nuclear condensation, growth suppression, and fragmentation Caspase-3/7/8/9 activation Overexpressed FasL Elevated p53 and BAX expression Mitochondria-dependent intrinsic and extrinsic pathway apoptosis Inhibition of AKT, p38, and NF-κB signaling pathways	Kang et al. (2022)
Cervical cancer	SiHa and HeLa cells	Elevated cleaved-caspase 3 and E-cadherin expression levels Reduced the number of invading cells and vimentin and N-cadherin protein levels Increased apoptosis Inhibited metastasis	Liao et al. (2023)
Prostate cancer	LAPC-4 and LNCaP cells	Combinatorial treatment synergistically augmented the antiproliferative impact Suppressed androgen receptor protein expression Suppressed both PI3K/AKT, STAT3 and AR pathways	Wang et al. (2015)
	Acid-resistant prostate cancer PC-3 cells	ROS-induced mitochondrial impairment Suppression of the PI3K/AKT/mTOR pathway	Lee et al. (2018)
	Acid-tolerant prostate cancer PC-3AcT cells	Addressed non-Warburg phenotype by inducing necroptosis through ROS-mediated CCN1 upregulation and mitochondrial damage	Lee Y. J. et al. (2020)
Bone cancer cells	MG63 and U-2 OS cells	Inhibited proliferation and colony formation, and induced apoptosis and S phase cycle arrest Impeded metastasis and facilitated cell cycle arrest and apoptosis by downregulating the expression of the enzyme heme oxygenase-1 (HMOX1)	Xu et al. (2023)
Nasopharyngeal carcinoma	5-8F cells.	Reduced invasiveness and motility Reduced expression levels of p-STAT3, EGFR phosphorylation, p-JAK2, and p-EGFR	Huang et al. (2023)
Melanoma cells	U87MG and T98G human glioma cell lines	Demonstrated notable cell growth, cell cycle arrest (G0/G1), and activation of apoptosis in both the cells	Maimaitili et al. (2017)

## 11 Combination chemotherapy

Combination chemotherapies are increasingly employed in cancer treatment to reduce toxicities and side effects by administering lower doses of the causative medications (Mayer and Janoff, 2007). Moreover, this approach has shown promise in overcoming treatment resistance (Shin et al., 2025). A previous study examined the effects of co-treatment with AG and DOX on the cytotoxic efficacy of DOX in MDA-MB-231 TNBC cells. AG augments the cytotoxic efficacy of DOX in AG-treated MDA-MB-231 cells by promoting p38-mediated AIF-dependent apoptosis and sustained p21 expression. In summary, AG may mitigate adverse effects and enhance the therapeutic efficacy of DOX (Lee K. S. et al., 2020).

Chemotherapy failure is frequently attributed to drug resistance, for which no viable therapeutic plan has been devised. Several studies have addressed drug resistance through the use of natural compounds. In MCF-DR and MDA-DR cells, DOX/AG induced G2/M arrest and necrotic cell death were caused by DNA damage and inhibition of DNA repair, which was attributed to increased DOX uptake and reduced cyclin D1 and MDR1 expression. Moreover, these findings demonstrate that DOX/AG-treated cells exhibit inhibited MAPK phosphorylation and reduced expression of MDR1, c-Jun, and cyclin D1, thereby inducing substantial necrotic cell death. The combination of AG and DOX has emerged as a viable therapeutic approach for DOX-resistant breast cancer (Lee et al., 2023).

Therapeutic utilization of AG is limited by its inadequate water solubility and rapid hydrolysis in the liver, gut, and plasma, which may limit its clinical application. Sialic acid (SA) binds to selectin receptors, which are overexpressed on the surface of tumor-associated macrophages. SA was coupled with octadecylamine (ODA) to synthesize SA-ODA, which was used to produce SA-functionalized nanoliposomes (SA-Lip) for targeted breast cancer therapy. The formulae were meticulously adjusted using the Box–Behnken design to increase the AG loading. The dimensions, entrapment efficiency, drug loading, and release characteristics of AG@SA-Lip were thoroughly examined and compared with those of conventional AG@Lip. AG@SA-Lip exhibited greater cytotoxicity and enhanced cellular internalization than AG@Sol and AG@Lip in the MCF7 and 4T1 cell lines. AG@SA-Lip had a minimal effect on the immune system. Consequently, SA-Lip exhibits significant potential as a delivery system for targeted administration of AG (Liu S. et al., 2024).

## 12 Clinical trials

AG has displayed significant therapeutic potential in several experimental settings, but very few clinical trials have shown promising results. Orally administered AG in gemcitabine-refractory pancreatic cancer, GBS-01, demonstrated high bioavailability with no dose-limiting toxicity. Nonetheless, among the 15 patients, not even a single patient achieved tumor response (Ikeda et al., 2016). Comprehensive information regarding these clinical trials is presented in Table 2.

**TABLE 2:** Reported clinical trials of arctigenin in various health implications.

Drug	Dosage	Duration	Participant condition	Findings	Reference
AG	250–500 mg q.d.	4 weeks	Healthy adult	Unavailable	NCT0303388 (clinical trial ID)
GBS-01	3–12 g equivalent AG q.d.	4 weeks	Advanced pancreatic carcinoma	Significant disease response (6.7%) and drug response rate (6.7%) No toxicity	Ikeda et al., 2016
Burdock sprout extract capsule	40 mg equivalent AG q.d.	12 weeks	Healthy adult	oxLDL suppression in AG administered healthy adult	Ito et al. (2021)

## 13 Conclusion and future perspective

Arctigenin has shown significant anti-inflammatory potential in regulating inflammation by inhibiting NF-κB activation and suppressing pro-inflammatory cytokines such as TNF-α and IL-6. AG possesses diverse mechanisms and a favorable safety profile, indicating its significant potential for the development of novel therapeutic techniques for chronic inflammatory disorders, including arthritis, neuroinflammation, and certain malignancies. To harness this potential, subsequent research must focus on clinical trials, pharmacokinetics, and dosage optimization in humans. Thus far, all clinical trials on AG have been conducted via the oral route and are considered convenient and safe. Nonetheless, significant biotransformation resulting from first-pass metabolism and inadequate water solubility impedes its practical application. Moreover, the ingestion of AG as a functional food may be feasible. The therapeutic potential of AG warrants recognition, and further efforts are necessary for its future clinical application to validate it as a potent and safe anticancer bioactive compound for cancer management.
